# Medical Assistance in Dying in Oncology Patients: A Canadian Academic Hospital’s Experience

**DOI:** 10.3390/curroncol29120739

**Published:** 2022-12-01

**Authors:** Tony Liu, Wei Liu, Aaron Leung, Sangyang Jia, Patsy Lee, Luke Liu, Adam Mutsaers, Sue Miller, Kimia Honarmand, Shiraz Malik, Melody Qu, Ian Ball

**Affiliations:** 1 Department of Medicine, Western University, London, ON N6A 5C1, Canada; 2 Division of Radiation Oncology, University of British Columbia, Vancouver, BC V5Z 4E6, Canada; 3 Department of Medicine, University of Manitoba, Winnipeg, MB R3E 3P5, Canada; 4 Michael G. DeGroote School of Medicine, Hamilton, ON L8P 1H6, Canada; 5 Department of Radiation Oncology, Odette Cancer Centre—Sunnybrook Health Sciences Centre, 2075 Bayview Avenue, Toronto, ON M4N 3M5, Canada; 6 London Health Sciences Centre Medical Assistance in Dying Program, London, ON N6H 1T3, Canada; 7 Department of Family Medicine, Western University, London, ON N6A 5C1, Canada; 8 Division of Radiation Oncology, Western University, London, ON N6A 5W9, Canada; 9 Department of Epidemiology and Biostatistics, Western University, London, ON N6A 5C1, Canada

**Keywords:** medical assistance in dying, euthanasia, end-of-life care, palliation

## Abstract

Background: Medical assistance in dying (MAID) was legislatively enacted in Canada in June 2016. Most studies of patients who received MAID grouped patients with cancer and non-cancer diagnoses. Our goal was to analyze the characteristics of oncology patients who received MAID in a Canadian tertiary care hospital. Methods: We conducted a retrospective review of all patients with cancer who received MAID between June 2016 and July 2020 at London Health Sciences Centre (LHSC). We describe patients’ demographics, oncologic characteristics, symptoms, treatments, and palliative care involvement. Results: Ninety-two oncology patients received MAID. The median age was 72. The leading cancer diagnoses among these patients were lung, colorectal, and pancreatic. At the time of MAID request, 68% of patients had metastatic disease. Most patients (90%) had ECOG performance status of 3 or 4 before receiving MAID. Ninety-nine percent of patients had distressing symptoms at time of MAID request, most commonly pain. One-third of patients with metastatic or recurrent cancer received early palliative care. The median time interval between the first MAID assessment and receipt of MAID was 7 days. Interpretation: Most oncology patients who received MAID at LHSC had poor performance status and almost all had distressing symptoms. The median time interval between first MAID assessment and receipt of MAID was shorter than expected. Only one-third of patients with metastatic or recurrent cancer received early palliative care. Improving access to early palliative care is a priority in patients with advanced cancer. Study registration: We received research approval from Western University’s Research Ethics Board (REB) with project ID number 115367, and from Lawson’s Research Database Application (ReDA) with study ID number 9579.

## 1. Introduction

Medical assistance in dying (MAID) was legislatively enacted in Canada in June 2016 and includes both euthanasia and Physician Assisted Suicide (PAS) [1,2].Euthanasia refers to the active administration by a MAID provider of medications to end a patient’s life, such as an injection of sedative agents and neuromuscular blockers [1,3]. PAS refers to the prescription or supply of a lethal drug or dose by a MAID provider for patient self-administration [1,3].

The current Canadian MAID eligibility criteria include being at least 18 years of age, being capable of making health care decisions for oneself, having a grievous and irremediable medical condition, making a voluntary request for MAID, and providing informed consent to receive MAID^2^. Safeguards currently in place include medical assessments by two independent practitioners, a signed written request for MAID before an independent witness, and the ability to withdraw request at any time, including final consent directly before MAID administration.

There have been over 21,000 MAID deaths in Canada from the time of legislation to 31 January 2021 [4]. In jurisdictions where forms of euthanasia or PAS are available, most patients request MAID because of a cancer diagnosis [5,6,7,8,9,10,11,12,13,14]. Likewise in Canada, approximately 70% of patients who received MAID had cancer [4]. Our group has previously reported on the MAID process and infrastructure at London Health Sciences Center (LHSC) [1]. However, the oncologic, symptom-related, and treatment-related characteristics of Canadian cancer patients who received MAID have not been carefully studied. Our goal was to describe the characteristics of oncology patients who received MAID at a Canadian tertiary care hospital.

## 2. Methods

We conducted a retrospective review of all patients who received MAID at LHSC from June 2016 to July 2020, and who had cancer as the primary reason for MAID eligibility. We excluded patients with previous cancer diagnoses who had completed curative-intent treatment more than five years earlier and had no evidence of cancer at time of MAID assessment. Patient identification, age, terminal diagnosis, dates of MAID requests, date of death, and cause of death were collected from a prospectively maintained database which included all patients who requested MAID at LHSC. Other data, including cancer diagnosis and stage, cancer therapies, symptoms at assessment, and specialist palliative care involvement were recorded retrospectively from clinical documents. We defined specialist palliative care physicians as those who provide palliative care as part of a dedicated palliative care service. Symptoms were considered distressing if they contributed to the patient’s desire for MAID, based on documentation by MAID assessors, the most recent oncology or palliative care note prior to MAID assessment, or both. Eastern Cooperative Oncology Group (ECOG) Performance Status was assigned based on clinical notes closest to the time of MAID request (within 4 weeks prior) and MAID receipt (within 2 weeks prior) [15]. This study was approved by Western and Lawson REB (REB 115367, 10 August 2021).

## 3. Results

Between June 2016 and July 2020, 561 patients requested MAID at LHSC, including 351 patients (63%) who had cancer as their terminal illness and 210 patients (37%) who had non-cancer diagnoses. During this time, there were roughly 6400 admissions and 24,000 consultations for cancer patients at LHSC. Ninety-two of 351 patients (26%) with cancer who requested MAID received MAID and were included in this study. For comparison, during the study period, 75 of 210 patients (36%) with non-cancer diagnoses who had requested MAID received MAID. This included 24 of 59 patients (41%) with cardiovascular or respiratory disease, 19 of 35 patients (54%) with neurological disease other than ALS, 6 of 16 patients (38%) with ALS, and 26 of 100 patients (26%) with other non-cancer diseases. These patients with non-cancer diagnoses were not included in this study.

Data on involvement of palliative care was available on 91/92 patients. The remaining patient had most oncologic care provided at a different hospital. Demographic information is reported in Table 1. Median age was 72 years old (interquartile range [IQR]: 65 to 80); 86/92 patients (93%) were 60 to 89 years of age. Of the patients with a reported place of residence, 47% of patients lived with their partner, 40% of patients lived alone, and 13% lived with family or roommates (*n* = 92).

**Figure 1:**
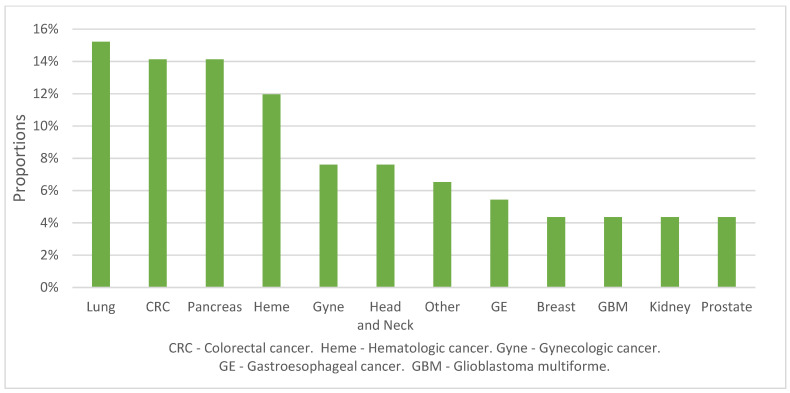
Cancer diagnosis distribution (*n* = 92).

**Figure 2:**
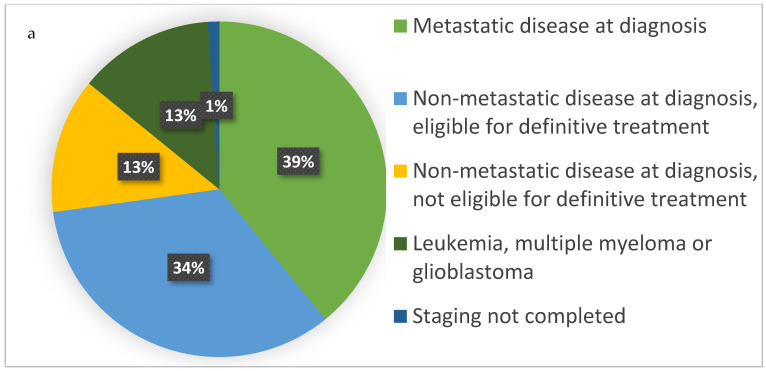

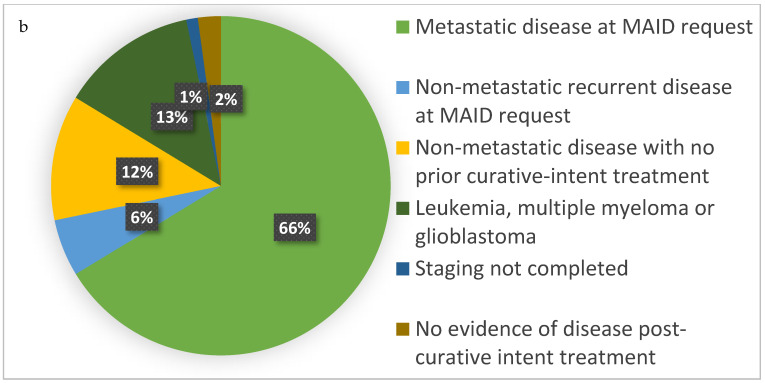
Cancer stage and status at diagnosis (**a**) and at MAID request (**b**) (*n* = 92).

The leading cancer diagnoses among patients who received MAID were lung (14/92, 15%), colorectal (13/92, 14%), and pancreatic (13/92, 14%) (Figure 1). At the time of diagnosis, 36/92 (39%) of patients had metastatic disease; 43/92 (46%) had non-metastatic disease; and 12/92 (13%) had multiple myeloma, leukemia, or glioblastoma, which are typically considered as incurable diseases without using a typical staging system (Figure 2a, *n* = 92). Of 31 patients who were eligible for curative-intent treatment at the time of diagnosis, 28 (90%) completed curative-intent treatment. At the time of MAID request, 72% of patients (66/92) had metastatic or locoregionally recurrent disease (Figure 2b). Among these patients, 67% (44/66) received at least one line of palliative-intent systemic (oncologic drug) therapy. Among patients who received systemic therapy, the median number of lines of palliative-intent systemic therapy was 1 (IQR: 1 to 2.75).

Two patients had no evidence of disease after receiving curative-intent treatment. One patient had pT4aN0M0 urothelial cancer involving the bladder and prostate and received MAID 2 months after cystoprostatecomy. Another patient had pT3N0 low rectal cancer treated with long course chemoradiation followed by low anterior resection. The patient experienced a number of operative complications and received MAID 8 months after low anterior resection.

**Figure 3:**
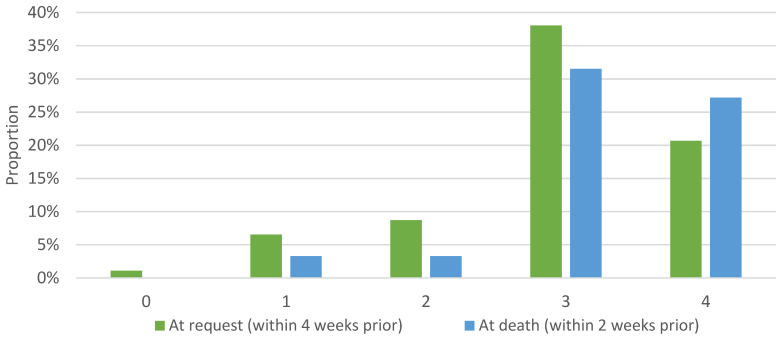
Eastern Cooperative Oncology Group Performance Status at time of MAID request (*n* = 69) and at time of death (*n* = 60). The Eastern Cooperative Oncology Group (ECOG) Scale of Performance Status is one measurement of how the disease impacts a patient’s daily living abilities. 0—Fully active, able to carry on all pre-disease performance without restriction. 1—Restricted in physically strenuous activity but ambulatory and able to carry out work of a light or sedentary nature, e.g., light house work, office work. 2—Ambulatory and capable of all selfcare but unable to carry out any work activities; up and about more than 50% of waking hours. 3—Capable of only limited selfcare; confined to bed or chair more than 50% of waking hours. 4—Completely disabled; cannot carry on any selfcare; totally confined to bed or chair. 5—Dead.

Of the 69 patients with reported ECOG scores within 4 weeks of MAID request, 54 patients (79%) had an ECOG score of 3 or 4. Of the 60 patients with reported ECOG scores within 2 weeks of receiving MAID, 54 patients (90%) had an ECOG score of 3 or 4 (Figure 3). Ninety-one of 92 patients (99%) had at least one distressing symptom; 12 patients had one distressing symptom, 29 patients had two distressing symptoms, and 51 patients had at least three distressing symptoms. Patients’ most frequently reported distressing symptoms at time of MAID assessment were pain (56, 61%), fatigue (35, 38%), dyspnea (25, 27%), weakness (23, 25%), nausea (19, 21%), lack of appetite (19, 21%), and declining functional status (15, 16%) (Table 2).

Twenty-one of 66 patients (32%) received early palliative care, as defined by palliative care within 8 weeks of diagnosis of metastatic or recurrent disease. The median number of days from diagnosis of metastatic cancer or disease recurrence to first palliative care consultation was 152 days (IQR: 20 to 570). Fifty-nine out of 91 patients (65%) received specialist palliative care prior to first MAID assessment. The median number of days from specialist palliative care consultation to first MAID assessment was 13 (IQR: 6 to 65). Another 20 patients received specialist palliative care following their first MAID assessment. The median number of days from MAID assessment to palliative care consultation was 1 (IQR: 0 to 18). Seventy-nine out of 91 patients (87%) received specialist palliative care in total. The median number of days from first palliative care consultation to receipt of MAID was 21 days (IQR: 12 to 80). The median time interval between the first assessment of MAID and receipt of MAID was 7 days (IQR: 3 to 13), reflecting that for many patients, the 10-day ‘reflection period’ requirement was waived. 

## 4. Discussion

Since its legalization in 2016, over 21,500 patients have received MAID in Canada, 70% of whom had cancer [4]. Existing studies on MAID have pooled data from patients with cancer and other terminal diagnoses. We found that among the 92 patients with cancer who received MAID, most patients had ECOG performance status of 3 or 4, 99% reported distressing symptoms, and approximately one-third received early palliative care.

Cancer patients choose MAID for a variety of reasons including physical or mental suffering, or the anticipation of it [16]. In this study, the most common distressing symptoms at the time of MAID request were pain, fatigue, dyspnea, weakness, and nausea. These symptoms have been shown to be associated with receipt of euthanasia in terminally ill cancer patients in the Netherlands [8]. In our experience, many patients also choose MAID in order to control the timing and circumstances of their deaths, as a mechanism to maintain personal dignity and autonomy.

The median interval from first MAID assessment to receipt of MAID was shorter than expected at 7 days. During this study period, waiving the 10-day reflection period between written MAID request and receipt of MAID was allowed if the patient was imminently dying or at risk of losing capacity as determined by two assessors. Thus, the short interval from first assessment to completion of MAID in our study is compatible with our finding that most patients had poor performance status at the time of MAID request. Of note, following the time period investigated in our study, a change in the Canadian MAID legislation occurred on 17 March 2021 that removed the 10 day waiting period for MAID in patients with a reasonably foreseeable natural death [17].

Over the last decade, several randomized trials have shown that specialty palliative care services provided concurrently with active cancer therapy leads to improved symptoms, mood, quality of life, and overall survival, and reduced emergency hospital admissions, hospital deaths, and caregiver distress [18,19,20,21]. Accordingly, The American Society of Clinical Oncology (ASCO) recommends early integration of palliative care services into the care of patients with metastatic malignancy, defined as within 8 weeks after diagnosis [19]. Although 65% of patients in our study received specialist palliative care prior to MAID assessment, palliative care involvement tended to be late in the patient’s disease course, with a median interval from diagnosis of metastatic or recurrent cancer to palliative care consultation of 22 weeks or 152 days. The median interval from first palliative care consultation to receipt of MAID was 21 days. Currently, no data exist on whether access to earlier palliative care impacts the frequency of MAID requests or deaths.

A systematic review examining barriers to palliative and hospice care utilization in older adults with cancer found that socio-demographic factors associated with lower likelihood of utilizing hospice or palliative care services included being male, unmarried, a racial minority, having lower median income and less education, and residing in rural areas [22]. Access to palliative care for cancer patients in Canada varies by geographic location, with rural and northern locations having reduced access to palliative care [23]. In non-metropolitan areas, there is lower availability of palliative care services, and this is one of the factors behind a lower referral rate of oncology patients to such services in Canada [24] and Australia [25].

Different models of early palliative care involvement have been reported and should be explored to improve access [26]. For example, studies have found that use of videoconferencing at a nearby telehealth facility or in patient homes, with an in-person nurse trained in symptom assessment, allowed for effective palliative care consultations that led to improved symptoms, significant cost savings and satisfaction among patients and referring physicians [27,28]. Advances in teleconference capabilities and incentives that have been enacted during the COVID-19 pandemic [29,30] may allow for more accessible care for oncology patients. Incorporating dedicated palliative care training into oncology training is another model that may improve access to early palliative care. For example, in rural Norway, physicians and nurses were each trained in oncology and palliative medicine [24].

Future work should include prospective studies which longitudinally evaluate patients with terminal cancer and their caregivers using validated questionnaires. An ongoing study at the University of Toronto seeks to better understand the various patient-centred physical, psychological, and social factors involved in the desire for death and MAID completion in Canadian cancer patients through validated questionnaires and qualitative interviews [31]. Given Canada’s cultural and ethnic diversity, it will be important to understand differences in attitudes and access to MAID and end-of-life care. Previous reports from the United States indicate that some minority groups, in particular African Americans, demonstrated greater needs for end-of-life care and that care provided was often incongruent with patient preferences [32]. Since the passage of Bill C-7 in March 2021, MAID assessors are now required to collect data regarding ethnic background with the permission of the patient, and this information is shared with Statistics Canada. It will be important to perform additional studies in Canada to determine if any marginalized groups have barriers to access culturally appropriate end-of-life care.

Strengths of this study include use of a large and detailed database that has been maintained since the MAID program’s inception. This study has limitations inherent to its retrospective nature, including limited reliability of symptom and performance status data.

## 5. Conclusions

Most oncology patients who received MAID at LHSC were 60–89 years of age, had poor performance status, and had distressing symptoms. Only one-third of patients with metastatic or recurrent cancer received early palliative care. Improving access to early palliative care is a priority in patients with advanced cancer.

## Figures and Tables

**Table 1:** Demographic data of patients with cancer who received MAID at LHSC.

Personal Data	Frequency	Proportions
Age		(*n* = 92)
<39	1	1%
40–49	4	4%
50–59	4	4%
60–69	27	29%
70–79	31	34%
80–89	21	23%
>90	4	4%
Sex		(*n* = 92)
Female	50	54%
Male	42	46%
Residence		(*n* = 92)
Partner	43	47%
Alone	37	40%
Children	8	9%
Parents	2	2%
Other	2	2%

**Table 2:** Incidence and proportion of reported symptoms at time of MAID assessment.

Distressing Symptoms Reported	Incidence	Proportion
pain	56	61%
fatigue	35	38%
dyspnea	25	27%
weakness	23	25%
nausea	19	21%
lack of appetite	19	21%
declining functional status	15	16%
dysphagia	9	10%
incontinence	5	5%
confusion	5	5%
depression	5	5%
other	27	29%

## Data Availability

The data presented in this study are available on request from the corresponding author. The data are not publicly available due to patient privacy and protection of personal health information.
